# MicroRNA-138 Abates Fibroblast Motility With Effect on Invasion of Adjacent Cancer Cells

**DOI:** 10.3389/fonc.2022.833582

**Published:** 2022-03-17

**Authors:** Saroj Rajthala, Himalaya Parajuli, Harsh Nitin Dongre, Borghild Ljøkjel, Kristin Marie Hoven, Arild Kvalheim, Stein Lybak, Evelyn Neppelberg, Dipak Sapkota, Anne Christine Johannessen, Daniela-Elena Costea

**Affiliations:** ^1^ The Gade Laboratory for Pathology, Department of Clinical Medicine, Faculty of Medicine, University of Bergen, Bergen, Norway; ^2^ Centre for Cancer Biomarkers (CCBIO), Faculty of Medicine, University of Bergen, Bergen, Norway; ^3^ Head and Neck Clinic, Haukeland University Hospital, Bergen, Norway; ^4^ Tannteam Private Dental Practice, Bergen, Norway; ^5^ Department of Oral Surgery, Institute of Clinical Dentistry, University of Bergen, Bergen, Norway; ^6^ Department of Oral Biology, University of Oslo, Oslo, Norway; ^7^ Department of Pathology, Haukeland University Hospital, Bergen, Norway

**Keywords:** cancer-associated fibroblasts, oral cancer, heterogeneity, motility, invasion

## Abstract

**Background:**

Recent studies have shown aberrant expression of micro-RNAs in cancer-associated fibroblasts (CAFs). This study aimed to investigate miR-138 dysregulation in CAFs in oral squamous cell carcinoma (OSCC) and its effects on their phenotype and invasion of adjacent OSCC cells.

**Methods:**

Expression of miR-138 was first investigated in OSCC lesions (*n* = 53) and OSCC-derived CAFs (*n* = 15). MiR-138 mimics and inhibitors were used to functionally investigate the role of miR-138 on CAF phenotype and the resulting change in their ability to support OSCC invasion.

**Results:**

Expression of miR-138 showed marked heterogeneity in both OSCC tissues and cultured fibroblasts. Ectopic miR-138 expression reduced fibroblasts’ motility and collagen contraction ability and suppressed invasion of suprajacent OSCC cells, while its inhibition resulted in the opposite outcome. Transcript and protein examination after modulation of miR-138 expression showed changes in CAF phenotype-specific molecules, focal adhesion kinase axis, and TGFβ1 signaling pathway.

**Conclusions:**

Despite its heterogeneous expression, miR-138 in OSCC-derived CAFs exhibits a tumor-suppressive function.

## Introduction

Carcinogenesis is a multistep process that is not only dependent on the intrinsic properties of cancer cells, but also determined by the host stroma or the surrounding tumor microenvironment (1–3). Fibroblasts are one of the major cell types in the stroma that can modulate the behavior of cancer cells at all stages of carcinogenesis, including metastasis (3, 4). In oral squamous cell carcinoma (OSCC), the role of cancer-associated fibroblasts (CAFs) for tumor progression has been demonstrated in *in vitro* cell culture studies (5–7) and *in vivo* animal studies (5). In clinical studies involving patient biopsies, CAFs have been associated to lymph node metastasis (8, 9) and poor prognosis in OSCC (8–12).

Apart from genetic alterations, epigenetic changes in cancer cells, including dysregulation of micro-RNAs (miRNAs) (13, 14) have also been widely recognized to have critical roles in cancer (15, 16). Dysfunction of miRNAs in stromal cells has been as well proven and shown to support further progression of transformed epithelial cells (17). Previous studies have shown miRNA deregulation in OSCC, their role in tumor progression, and their possible use as diagnostic and prognostic biomarkers, as well as therapeutical targets (18–20). However, few studies investigated consequences of alterations of miRNAs in tumor stroma for tumor progression in general, and for OSCC in particular.

In a recent study that involved miRNA profiling of primary cultures of OSCC-derived CAFs and normal oral fibroblasts (NOFs), we found altered expression of twelve miRNAs in CAFs (21). When coupled with the results of our previous transcriptomic study on the same strains of OSCC-derived CAFs and NOFs that found integrin α11 upregulated in CAFs of OSCC (5), miR-204 and miR-138 were identified by *in silico* prediction tools as possible upstream regulators of integrin α11. We further showed that indeed integrin α11 is a direct target of miR-204 and that miR-204 plays an anti-invasive role in OSCC (21), but we did not investigate miR-138 for its role in OSCC.

MiR-138 has been found to be downregulated in many cancer types, including OSCC, and it was therefore coined as a tumor suppressor (22). On the other hand, it has been linked to tumor progression and recurrence in glioblastoma (23). In a previous study on OSCC (42 cases), miR-138 was shown to have a decreased expression in OSCC compared to adjacent normal mucosa (24). In another study (*n* = 35), its expression was relatively higher in about half of OSCC when compared to normal oral mucosa (25). *In vitro* studies showed anti-proliferative and anti-invasive role for miR-138 when expressed in OSCC cells (26), and it was suggested to have a role in suppressing cancer stemness (27).

While the above-mentioned studies illustrate the role of miR-138 in several cancers when dysregulated in either whole tumor tissue or only in the epithelial compartment, studies on the consequences of altered miR-138 expression in stromal fibroblasts for tumor progression are, to our best knowledge, non-existent. This study aimed to investigate the role of miR-138 dysregulation in CAFs on OSCC progression.

## Materials and Methods

### Patient Material

For miRNA *in situ* hybridization, formalin-fixed paraffin-embedded (FFPE) tissue blocks from OSCC lesions (*n* = 53) were used from the diagnostic archive of the Department of Pathology of Haukeland University Hospital. Clinical data were obtained from the Electronic patient journal (*n* = 38, **Supplementary Table S1**). Clinical–pathological correlations of the cohort were in line with a previous finding from larger cohorts of OSCC, indicating that the cohort could be used for preliminary biomarker analysis. Multi-variate Cox regression found tumor stage, age, and gender as independent predictors of survival with increased death risk for late tumor stage HR: 2.22 (1.03–4.76), age group above 65 HR: 2.28 (1.04–5.00), and male group HR: 4.29 (1.44–12.79), respectively (**Supplementary Figure S1**). Similarly, tumor stage was as independent predictor of recurrence-free survival, with increased risk for recurrence for the late stage group HR: 3.662 (1.18–11.31). Controlled for tumor stage, tumor site predicted higher risk of recurrence for OSCC lesions involving gingiva compared to tongue HR: 3.19 (1.985–10.322).

For isolation of paired cancer-associated and normal fibroblasts, fresh tissues from the tumor and healthy mucosa (more than 1 cm away from the OSCC lesion) from OSCC patients (*n* = 6) were collected at the time of the surgical excision. Non-matched CAFs were also isolated from additional primary OSCC lesions (*n* = 9) and normal oral fibroblasts (NOFs) from oral mucosa of non-cancer individuals (*n* = 10). Only HPV negative primary tumors without any prior therapies were included in the study. Informed consent was obtained in all cases. Ethical approval for the study was obtained from the regional ethical committee (REKVest 2010/481).

### *In Situ* Hybridization and miRNA Semi-Quantification

ISH of OSCC tissues was performed as described earlier (28). In brief, 3-µm sections of formalin-fixed and paraffin-embedded tissues were deparaffinized in xylene, rehydrated in series of 99%, 96%, and 70% alcohol concentration, and epitope retrieved with 15 μg/ul Proteinase K (90000; Exiqon, Denmark) solution at 37°C for 10 min. At 53°C, sections were pre-hybridized with ISH buffer (90000; Exiqon; Denmark) for 30 min, and then incubated for an hour with digoxigenin (DIG) labeled miR-138-5p specific oligonucleotides (612107-360; Exiqon, Denmark). Thereafter, tissues were washed with decreasing concentrations of saline-sodium citrate buffer (S66391L; Sigma, USA), and then blocked with 2% sheep serum (013-000-121; Jackson ImmunoResearch, USA) in 1% bovine serum albumin. Subsequently, tissues were incubated with alkaline phosphatase (ALP)-linked anti-DIG Fc fragments (1:400; 11093274910; Roche, Germany) overnight at room temperature. The tissues were thoroughly washed and then incubated with ALP substrate-Nitro blue tetrazolium chloride/5-Bromo-4-chloro-3-indolyl phosphate (NBT-BCIP) (11681451001; Roche, Germany) at 30°C for 2 h. Levamisole (X3021; Dako, USA) was mixed with the substrate to block endogenous ALP activity. Finally, tissues were counterstained with nuclear fast red. A scramble oligonucleotide without target and small nuclear RNA-U6 were used as negative and positive controls, respectively.

miR-138 staining in OSCC sections were scored negative (0) or 1–4 according to increasing stain intensity by experienced pathologists.

### TCGA Data Analysis

TCGA miR-138 expressions from TCGA miRNA sequence data (*n* = 488) and clinical data for head and neck squamous cell carcinoma cohort (*n* = 528) were accessed from the Firebrowse database version 2016_01_28 (http://www.firebrowse.org). The same cohort contained miR-138 data for normal human oral mucosa (NHOM). After exclusion of HPV-positive, non-oral cancers cases and cases with history of neoadjuvant treatment, 277 oral cancer cases (alveolar ridge: 13; base of tongue: 11; buccal mucosa: 30; floor of mouth: 56; hard palate: 5; lip: 3; unspecified region in oral cavity: 62; tongue: 112; oropharynx: 7) with miR-138 data remained. Using the same exclusion criteria, out of 45 cases, only 25 NHOM cases remained. Of the 25 NHOM cases, 24 were matched to OSCC lesions (from the same patient).

### Cell Culture

Isolated CAFs and NOFs from OSCC patients and healthy donors were cultured in Dulbecco’s Modified Eagle’s Medium (DMEM; D6429, SIGMA) supplemented with 10% heat-inactivated newborn calf serum (NBCS; 31765068, GIBCO). OSCC cell lines UK1 (29) and Luc4 (30) were grown in DMEM/Nutrient Mixture F-12 Ham medium (D8437, Sigma) supplemented with 10% NBCS, 1× Insulin-Transferrin-Selenium (41400-04, Thermofisher Scientific), 0.4 μg/ml hydrocortisone (H0888, Sigma), 50 μg/ml L-ascorbic acid (A7631, Sigma), and 10 ng/ml epidermal growth factor (E9644, Sigma). All cell lines were propagated in humidity incubator at 5% CO_2_ and 37°C temperature and regularly tested for mycoplasma contamination.

### miRNA Modulation in Fibroblasts and Proliferation Assay

Each of the 1 × 10^6^ NOFs and CAFs was reverse transfected with mimics and inhibitors of miR-138-5p (C/IH-300605; Dharmacon), and the respective controls (mimic: CN-0010000-01; inhibitor: IN-001005-05; Dharmacon) at 50 nM concentration using LipofectamineTM 3000 Transfection Reagent (L3000015; Invitrogen, USA) following the manufacturer´s protocol. Forty-eight hours after the transfection, the cells were either harvested for molecular profiling or subjected to further functional studies. In order to see the effect of miR-138 on fibroblast proliferation, 1 × 10^4^ NOFs or CAFs were reverse transfected with mimics and inhibitors of miR-138-5p and the respective controls in quadruplicates in 24-well plates. After 48 h, the cells were Trypsin EDTA detached and counted using Trypan blue in Invitrogen Countess automated cell counter.

### RNA and miRNA Isolation

Total RNA was isolated using mirVana miRNA isolation kit (AM1560, mirVana). In brief, sub-confluent CAFs and NOFs in monolayer cultures were washed with phosphate buffered saline (PBS), lysed with Lysis/Binding buffer and Phenol : Chloroform extracted. Subsequently, RNA was captured in glass fiber filter column and eluted in elution solution. The purity and quantity of the RNA was measured using NanoDrop^®^ ND-1000 Spectrophotometer (Nanodrop Technologies; USA). Total RNA and enriched small RNAs were stored at −80°C until use.

### Reverse Transcription

Total RNAs were reverse transcribed to cDNAs using miRNAs specific primers using TaqMan MicroRNA Reverse Transcriptase kit (4366596, Applied Biosystem). In brief, 10 ng of total RNA was mixed with dNTPs, reverse transcription buffer, RNase inhibitor, and miRNA specific primer and reverse transcribed to a final reaction mixture of 15 μl. Thereafter, the reaction mixture was subjected to thermal cycle at 16°C for 30 min, 42°C for 30 min, and 85°C for 5 min. For mRNA quantification, total RNA was reversed transcribed using the Taqman Reverse Transcription kit (N8080234, Applied Biosystems). In brief, 100 ng of total RNA was mixed with reverse transcription buffer, MgCl_2_, dNTPs, random hexamer, RNase inhibitor, and reverse transcriptase to a final volume of 25 μl with RNase-free water. cDNA synthesis was performed at 20°C for 10 min, 48°C for 30 min, and 90°C for 5 min.

### Quantitative Real-Time Polymerase Chain Reaction

The expression of miRNAs and gene transcripts were quantified using Taqman assays in ABI Prism 7900 HT sequence detector system (Applied Biosystems). The PCR reaction volume was set to 10 μl for each well in 384-well plates. The PCR was then run at 50°C for 2 min, 95°C for 10 min, and for 40 cycles at 95°C for 15 s and 60°C for 1 min. Each sample was run in triplicate. mRNA expression was normalized to the housekeeping gene GAPDH, and miRNA expression was normalized to the expression of RNU48. Taqman assays used are listed in **Supplementary Table S2**.

### Western Blot

Semi-quantitative assessment of proteins of interest was performed using Western blot technique. In brief, protein lysates of fibroblast culture, 48 h post transfection of miR-138 mimics, inhibitors, and controls, were resolved in NuPAGE Novex 10% Bis-Tris Protein Gel (NP0303, Invitrogen) in NuPAGE MOPS SDS Running Buffer (NP0001, Invitrogen) at 160 V for 90 min and were transferred to PVDF membrane (10600069, GE Healthcare) in NuPAGE transfer buffer (NP0006, Invitrogen) at 40 V for an hour. Thereafter, PVDF membrane was blocked with 5% non-fat dry milk or 3% BSA in TBS-tween buffer for half an hour and incubated with primary antibody overnight at 4°C. The following day, PVDF membrane was thoroughly washed with TBS-tween, incubated at room temperature with secondary antibody tagged with horseradish peroxidase and thoroughly washed again. Finally, bands of proteins were visualized using SuperSignal West Pico Chemiluminescent Substrate (34080, Thermofisher) using Image Reagder LAS 1000 (Fujifilm), and protein band intensity in the captured images was quantified using ImageJ using Gel commands. GAPDH was used as loading control. Antibodies used in this study are listed in **Supplementary Table S3**.

### miRNA Dual Luciferase Target Reporter Assay

3’UTR sequence of ITGA11 (NM_001004439.1) was retrieved from the UCSC genome browser (http://genome.ucsc.edu) (31). A plasmid vector with luciferase upstream of 3’UTR and renilla as a control reporter was designed and purchased from Vector Builder. Position 1-1355 of ITGA11 3’UTR length harboring miR-138 binding site (724–730: CACCAGC)→ was inserted into the vector. For a control vector, non-complimentary mutant sequence GTGGTCG was introduced to miR-138 binding site. Transfection mix of plasmid DNA (250 ng per well in 24-well plates) and miR-204 mimic (calculated at 50 nM concentration in cell culture medium) was prepared using LipofectamineTM 3000 Transfection Reagent. Required volume of transfection mix and 5 × 10^5^ CAFs were mixed in each well, and the cells were maintained in the culture chamber for 48 h. Thereafter, the cells were harvested, and luciferase activity was measured using Dual luciferase detection system (E1910, Promega) following the manufacturer’s protocol using a Tecan Infinite M200PRO luminometer.

### Fibroblast Migration in Collagen Gel Assay

On the first day of the experiment, 1×10^5^ UK1 cells were plated in 24-well plates. The next day, collagen type I matrices were prepared by mixing collagen type I (354236, Corning), DMEM, NBCS and reconstitution buffer (2.2 g NAHCO_3_ + 0.6 g NAOH + 4.766 g HEPES 100 ml water) at a volume ratio of 7:1:1:1 on ice. The collagen matrix (250 µl per well) was pipetted into 0.4-µm 24-well Corning transwell inserts (CLS3413, Sigma) and allowed to gel at 37°C in an incubation chamber. Two hours later, the gels were layered on the top with 250 µl of 5×10^5^/ml fibroblasts modulated with miR-138. The co-culture system was maintained at 37°C for 5 days.

### Collagen Contraction Assay

Ninety-six-well plates were blocked with 2% BSA overnight at 37°C in an incubation chamber. Forty-eight hours post miRNA modulations, fibroblasts were suspended in collagen type I matrix prepared as described above at a density of 5×10^5^ cells/ml. Subsequently, 100 µl of fibroblast-collagen matrix was dispensed into each well and allowed to gel for 90 min. The gels were then gently dislodged from the surface of culture plate using 100 µl of DMEM medium. The gels were maintained at an incubation chamber and the change in gel dimension was measured at different time points.

### Fibroblast-OSCC Cell 3D Organotypic Co-Culture

3D co-culture models mimicking local invasion of OSCC cells into subjacent connective tissue were constructed by layering OSCC cells on top of a fibroblast-embedded collagen I matrix. In brief, either CAFs or NOFs at a density of 2.5 × 10^5^ cells/ml were suspended in the matrix of collagen type I prepared as above, on ice. Seven hundred microliters of the CAF- or NOF-populated collagen suspensions was pipetted into each well in 24-well plates and allowed to polymerize in a humidified incubator at 37°C. After 2 h, each well was gently added with 1 ml of complete DMEM medium to allow the cells to grow until the next day. The next day, 5 × 10^5^ cells of UK1 or Luc4 were added on the top of the fibroblast gel. A day after, the gels were transferred to a metal grid layered with a filter paper and grown on air-medium interface in DMEM : Ham´s F12 Nutrient mixture (31765068, Thermofisher) supplemented with insulin-transferrin-selenium, hydrocortisone, and L-ascorbic acid as above, but NBCS was replaced with 0.1% bovine albumin fraction (V15260-037, Thermofisher). The gels were cultured for the next 10 days. Medium was changed at each alternative day.

### Quantification of Invasion of OSCC Cells in 3D-Organotypic Models

FFPE-embedded organotypic tissues were cut into 5-μm sections and stained with hematoxylin and eosin. Images of the stained tissues were captured at 20× objective using a slide scanner (Hamamatsu NaNoZoomer-XR, Shizuoka, Japan) and the invasion depth of OSCC cells was measured using NDP.view2 (Hamamatsu, Japan). Depth of invasion was defined as the vertical distance from the reconstructed basement membrane (horizontal line along the non-invading cells) to the deepest invaded OSCC cells in the respective point. Twenty measurements of invasion at 50-µm distance along the tissue were taken and averaged. The non-uniform thick or tapered 100-µm ends of the 3D organotypic tissues were excluded from measurements.

### Statistical Analysis

Student´s unpaired or paired *t*-test or one-way ANOVA was used to examine significant differences in means in between two or more than two groups, respectively. Where data did not show a normal distribution (D´Agostino & Pearson test; *p* > 0.05), non-parametric comparisons (Wilcoxon for paired comparison and Mann–Whitney for unpaired comparison between two, and Kruskal–Wallis for unpaired comparison among groups) were carried out to determine significant difference in median expression. All analysis was performed using GraphPad Prism Version 7. For statistical analysis, the OSCC cohort was categorized into a negative or a positive miR-138 staining group or a low-no (0–1) and a high (2-4) miR-138 staining group. Overall survival (OS) and recurrence-free survival (RFS) analysis for clinicopathological parameters and miR-138 expression (positive and negative staining group) was carried out using log-rank test (Mantel–Cox). Clinicopathological parameters were further tested with multivariate Cox’s proportional regression to identify independent predictors of OS and RFS. Pearson’s chi-square test was carried out to determine the association of miR-138 status [positive (*n* = 31) versus negative (*n* = 7); low-no miR-138 (*n* = 33) versus high miR-138 (*n* = 4)] with clinicopathological parameters. Survival and association tests were carried out using IBM SPSS Statistics Version 25.

## Results

### miR-138 Was Expressed in a Subset of OSCC Lesions Only and Showed a Marked Heterogeneity in Both Epithelial and Stromal Compartments

**Figure 1:**
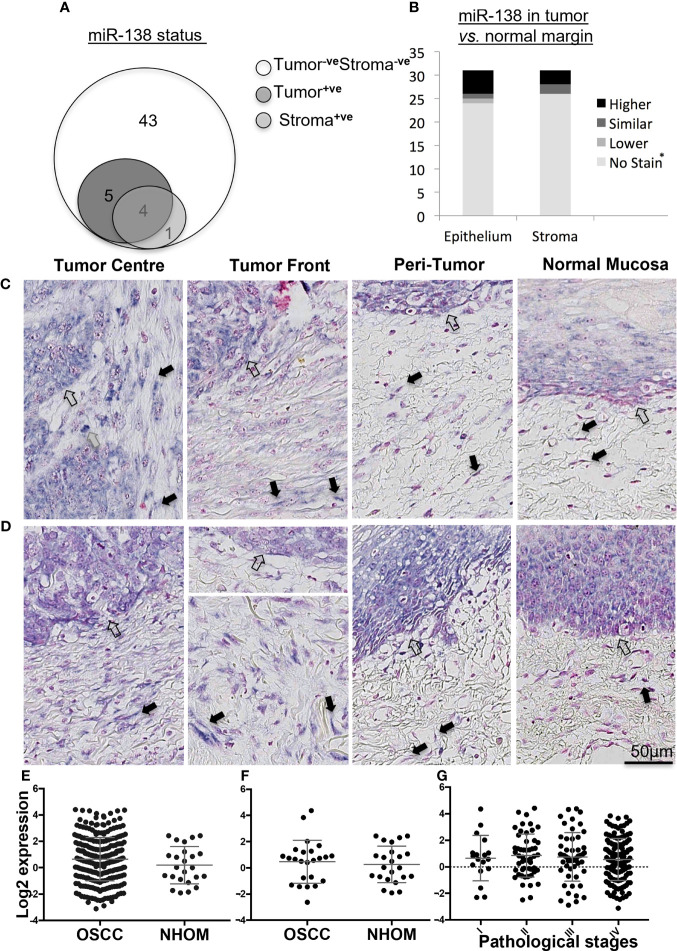
Expression of miR-138 in OSCC. **(A)** Venn diagram showing distribution of OSCC cases according to miR-138 staining positivity in the epithelial and tumor-associated stroma regions. **(B)** Stacked bar plot comparing the miR-138 expression in epithelial and stromal compartments in the OSCC lesions compared to peritumoral/normal margins. *^-ve^Stain in both tumor and adjacent normal/peritumor area. **(C)** Higher miR-138 expression in cancer cells in tumor center (TC) and tumor front (TF) compared to adjacent peritumor (PT) and normal (TN) areas. **(D)** Higher expression in PT region compared to TC and TF. Lymphocytes, fibroblasts, and epithelial/malignant compartment in gray, black, and unfilled arrows, respectively. **(E)** TCGA miR-138 expression in between OSCC and matched NHOM, **(F)** OSCC and unmatched NHOM, and **(G)** among pathological stages in OSCC. Unpaired *t*-test, paired *t*-test, and one-way ANOVA, respectively, for **(C–E)**.

Epithelial expression of miR-138 was observed in 17% (*n* = 9) of OSCC cases while stromal expression was detected in 9.4% (*n* = 5) of the cases. In 7.5% (*n* = 4) of the cases, miR-138 was expressed in both tumor and stromal compartments (**Figure 1A**). Pearson chi-square test showed that miR-138 staining positivity associated with lower depth of invasion: <4 mm (*p* = 0.05) and relatively higher miR-138 expression (epithelial and stromal) associated with lower OSCC recurrence (*p* = 0.03). Expression of miR-138 did not associate to any other clinical or pathological parameters, including tumor stages. Epithelium of NHOM controls showed miR-138 expression in 50% of cases (*n* = 3) while no expression was observed in subjacent normal stroma. Compared to histologically normal peritumoral epithelium, miR-138 expression in tumor epithelium was increased in 16.1% (*n* = 5) and decreased in 3.2% (*n* = 1) cases. Stromal miR-138 expression was increased in tumor stroma compared to respective stroma in normal/peritumor regions in 9.7% (*n* = 3) cases, while no difference was observed for the rest (**Figure 1B**). When expressed in tumor stroma, miR-138 was localized in cells with fibroblast morphology and in a sub-population of lymphocytes (**Figures 1C, D**).

Analysis of the TCGA data showed no significant difference in miR-138 expression between whole OSCC lesions compared to NHOM (**Figure 1E**). A difference in miR-138 expression was still not observed when the subset of OSCC cases was compared to their matched NHOM (**Figure 1F**). No significant difference in miR-138 expression among overall pathological tumor stages (I–IV) was found (**Figure 1G**).

### Fibroblasts From OSCC Lesions Displayed a Heterogeneous Expression of miR-138

**Figure 2:**
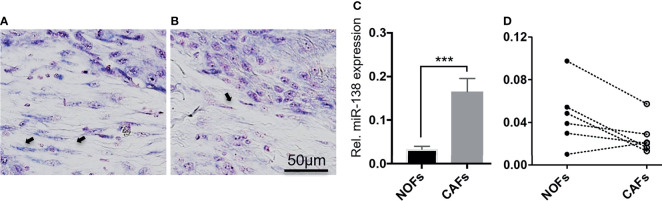
Heterogenous expression of miR-138 in cultured fibroblasts from OSCC lesions and normal mucosa. **(A, B)** Differential miR-138 expression in CAFs from different regions of tumor center. Fibroblasts are marked with arrows. **(C)** miR-138 expression in non-matched CAFs and NOFs. **(D)** miR-138 expression in matched NOFs and CAFs. Each pair is connected by dotted lines. ***p < 0.001.

Positive and negative miR-138-stained fibroblasts were observed to co-exist nearby the stroma of a subset of OSCC lesions (**Figures 2A, B**). qRT-PCR profiling of miR-138 expression in CAFs isolated from OSCC lesions and NOFs isolated from oral mucosa of non-related healthy, non-cancer individuals showed significantly higher expression in CAFs by 4.52-fold (**Figure 2C**). However, profiling of miR-138 in matched CAFs and NOFs showed a marked heterogeneity; out of the six matched pairs, miR-138 expression was higher in CAFs, then in NOF only in one matched pair, similar in two pairs, and lower in three pairs (**Figure 2D**).

### Ectopic Expression of miR-138 Decreased Fibroblast Proliferation and Expression of Several CAF-Related Markers, but Not of ITGA11

**Figure 3:**
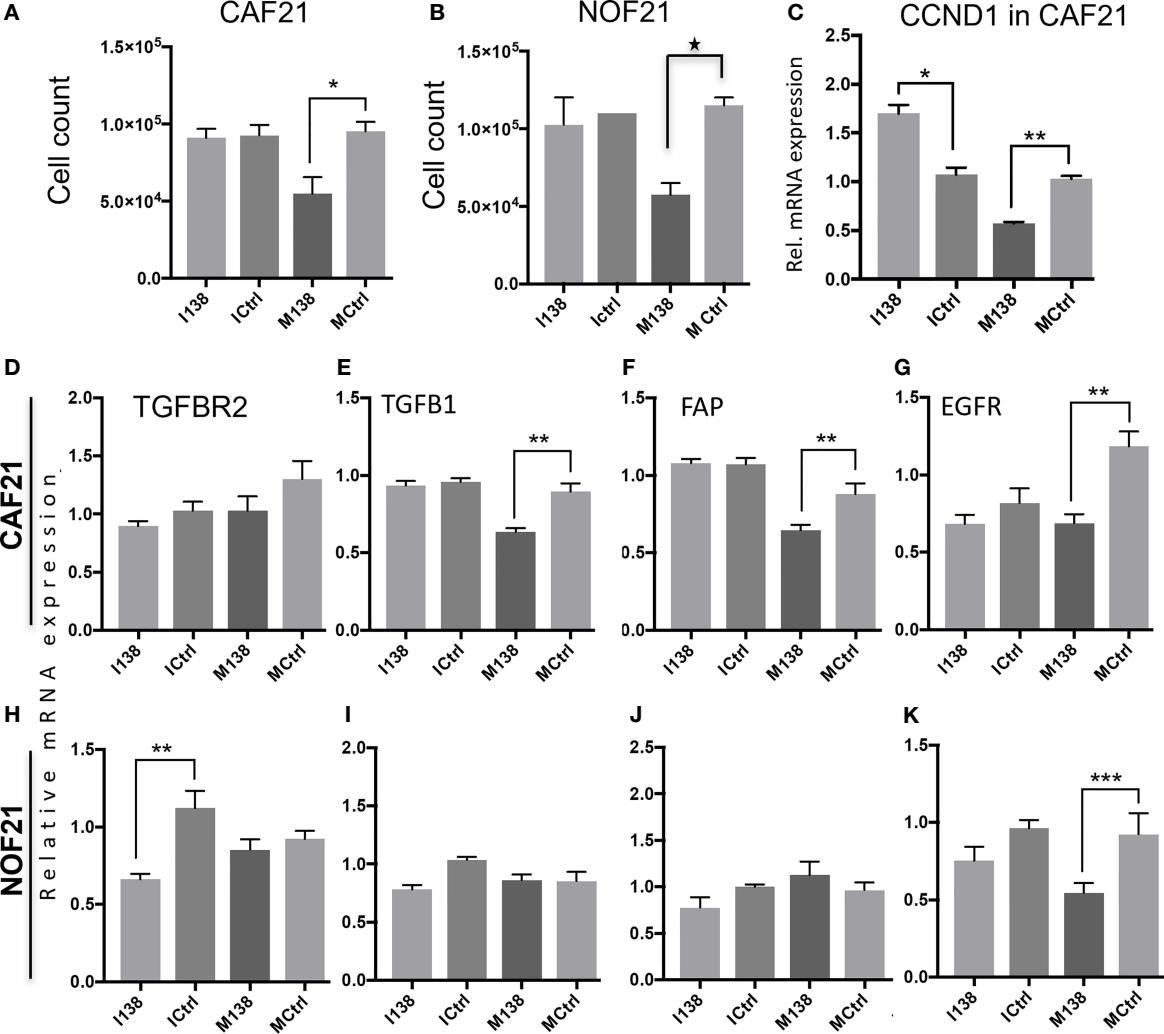
Fibroblasts’ proliferation and expression of CAF markers in monolayer culture following miR-138 modulation. Proliferation of **(A)** CAFs and **(B)** NOFs, **(C)** regulation of CCND1 transcript in CAFs, and **(D–K)** other transcripts related to the CAF-phenotype (TGFβ1, TGFBR2, and FAP) and EGFR in CAFs and NOFS 48 h post miR-138 modulation. * Significant; unpaired *t*-test *p* < 0.05. I138: inhibition of endogenous miR-138. M138: mimicking of miR-138 expression. Ictrl and MCtrl: respective controls for inhibitors and mimics. **p < 0.01, ***p < 0.001.

Ectopic expression of miR-138 (modulation of miR-138 expression in fibroblasts by use of mimics and inhibitors is presented in **Supplementary Figure S2**) resulted in significantly reduced proliferation of both CAFs and NOFs compared to mimic controls in monolayer culture (**Figures 3A, B**). Reduced proliferation of fibroblasts was accompanied by significant reduction in expression of CCND1 transcript (**Figure 3C**). However, despite upregulation of CCND1 following inhibition of miR-138, a change in fibroblast proliferation was not observed between the target and control group. Modulation of miR-138 expression also induced significant changes in expression of several CAF-related molecules (TGFBR2, TGFβ1, and FAP) and EGFR (**Figures 3D**–**K**).

**Figure 4:**
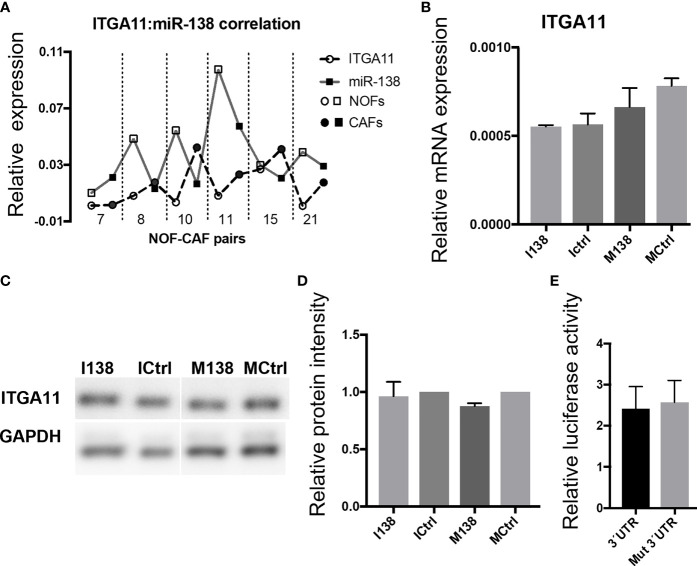
miR-138 does not target ITGA11. **(A)** Graph showing an inverse correlation between miR-138 and ITGA11 levels in the fibroblasts. **(B)** Modulation of miR-138 expression did not result in alterations of ITGA11 expression at mRNA or **(C, D)** protein levels in CAF. **(E)**; Gene reporter assay showing no difference in the expression of ITGA11 or mutant transcripts.

qRT-PCR profiling of miR-138 and ITGA11 transcripts in cultured fibroblasts showed an inverse correlation between their expression (**Figure 4A**). However, modulation of miR-138 expression did not result in alterations of ITGA11 expression at mRNA or protein levels (**Figures 4B**–**D**). Gene reporter assay showed no difference in expression of ITGA11 or mutant transcripts (**Figure 4E**), indicating that miR-138 does not target ITGA11.

### Ectopic Expression of miR-138-5p Induced a Change in Fibroblasts’ Morphology and Decreased Their Motility and Collagen Contraction Ability

**Figure 5:**
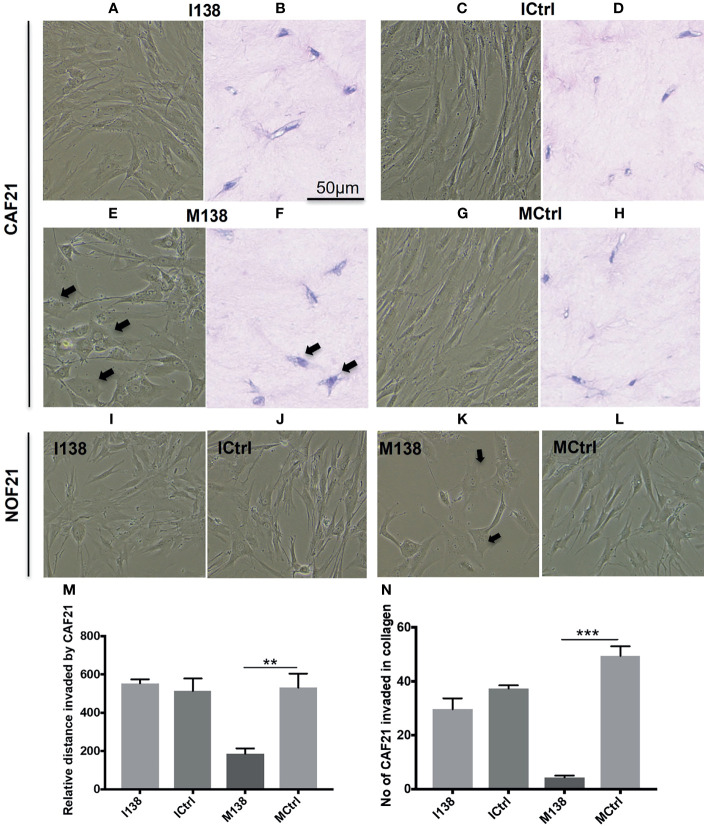
Altered fibroblast morphology and migration following miR-138 modulation in CAF monolayer and 3D-collagen matrix. **(A, C, E, G, I–L)** Phase contrast images for cells in monolayer taken 48 h post transfection of mimic and inhibitor of miR-138, and respective mimic and inhibitor control (50 nM). **(B, D, F, H)** Images of HE-stained sections of fibroblasts in 3D-collagen matrix post miR-138 modulation **(M, N)**. Effect of miR-138 modulation in CAFs, in its migration (invasion) ability in collagen gel matrix. M138—fibroblasts treated with miR-138 mimics. (*n* = 4–6, unpaired *t*-test, **p < 0.01, ***p < 0.001). I138—fibroblasts treated with miR-138 inhibitors of miR-13. Ictrl and MCtrl—respective controls for inhibitors and mimics.

**Figure 6:**
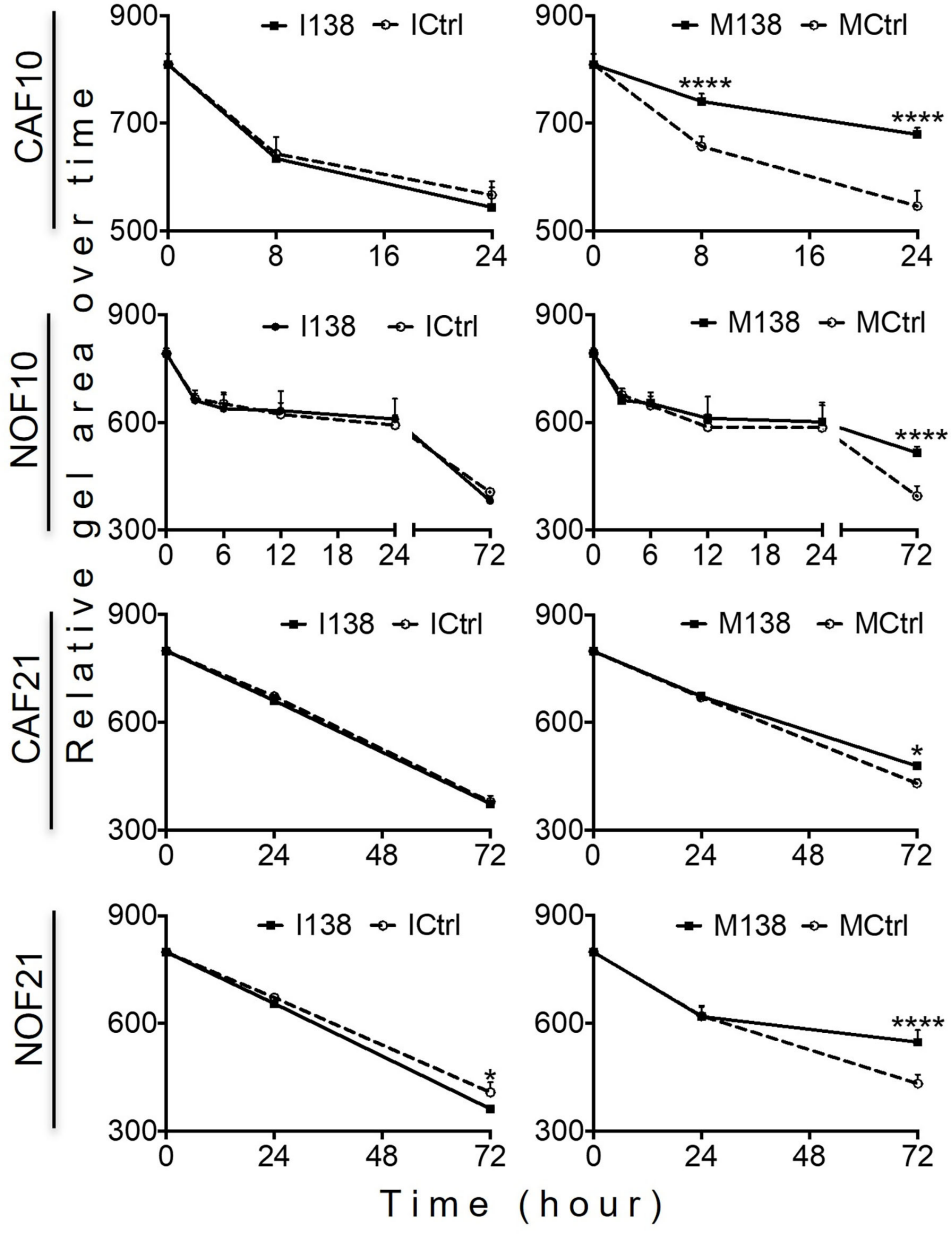
Altered miR-138 expression modulates the ability of fibroblasts to contract collagen gels: Collagen I contraction by CAFs and NOFs over time. *n* = 4–6; one-way ANOVA; mean ± SD; *p < 0.05, ****p < 0.0001.

Increasing miR-138 expression in fibroblasts (both CAFs and NOFs) changed their cellular morphology from an elongated, slender shape (**Figures 5G–H, L**) to a flattened, stellar shape and bigger size, compared to mimic controls (**Figures 5E, F, K**). Fibroblasts’ motility in 3D collagen I gels towards OSCC cell line UK1 was significantly impaired following transfection with miR-138 mimics; the number of fibroblasts that migrated inside the collagen gels and the distance crossed were significantly reduced in fibroblasts transfected with mimics, compared to controls (**Figures 5M, N**). Additionally, mimicking increased miR-138 in CAFs and NOFs and significantly reduced collagen contraction ability by both CAFs and NOFs, and reversing miR-138 expression by using inhibitors resulted in the opposite effect (**Figures 5A–D** and **6**).

### miR-138 Expression in Fibroblasts Decreased Invasion of Suprajacent OSCC Cells

**Figure 7:**
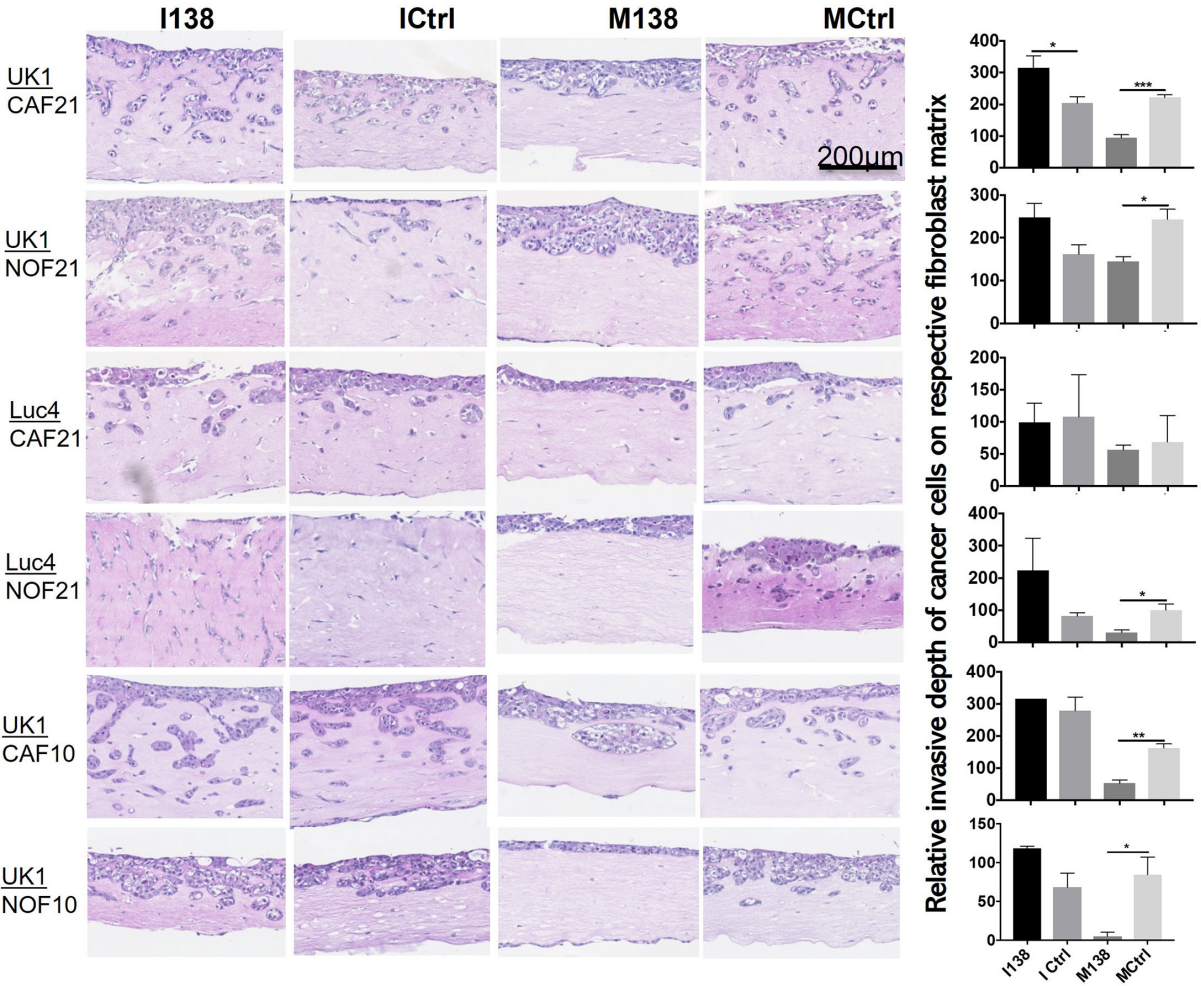
Altered miR-138 expression in fibroblasts modulates invasion of adjacent OSCC cells. HE images of 3D organotypic co-culture depicting invasion of OSCC cells in fibroblast-collagen matrix. Measurement of corresponding invasion distance by OSCC cells on the right. *p < 0.05, **p < 0.01, ***p < 0.001.

In order to study the role of miR-138 expression in CAFs on tumor invasion or progression, miR-138 expression was altered in CAFs and NOFs prior to their co-culture with the established OSCC cell lines UK1 and Luc4 in 3D-organotypic models. Inhibition of miR-138 in both CAFs and NOFs increased invasion by OSCC cell lines UK1 and Luc4, while transfection of both fibroblasts with mimics of miR-138 significantly decreased or almost completely neutralized invasion of both UK1 and Luc4 (**Figure 7**).

### Pathway Focus Analysis of Molecules Targeted by miR-138 Indicates Alterations in the Focal Adhesion Pathway

**Figure 8:**
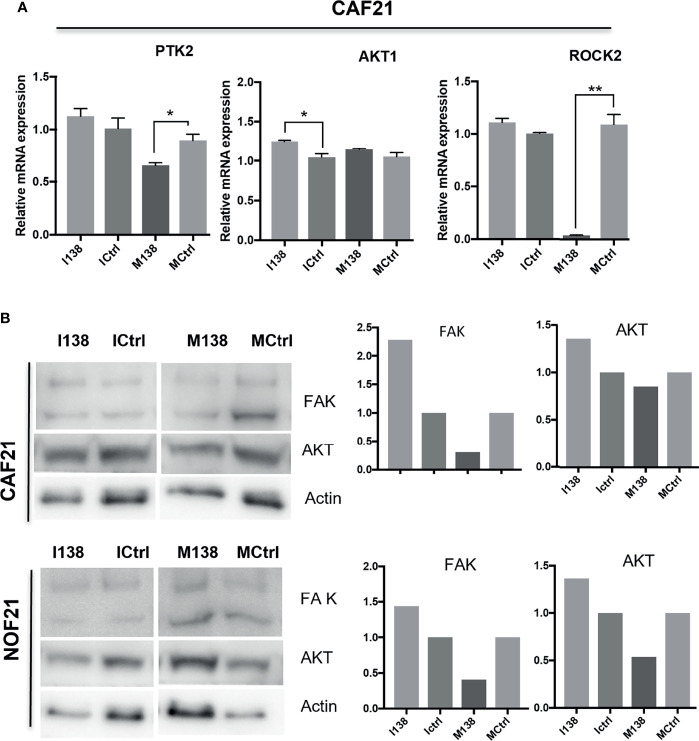
miR-138 expression and investigation of focal adhesion kinase (FAK) axis in CAFs. Alterations in **(A)** mRNA and **(B)** protein expression of several molecules of the FAK axis following miR-204 modulation in CAFs and NOFs. **(B)** Western blot images and the semi-quantification of protein blots with ImageJ. *p < 0.05, **p < 0.01.

Pathway analysis of genes targeted by miR-138 using miRTarBase database release 7 (32) identified several miR-138 targeted pathways, including focal adhesion and TGF-β1 pathways (**Supplementary Table S4** and **Figure S3**). Since FAK, AKT, ROCK, and CCND1 have been previously proven by luciferase gene reporter assays to be direct targets of miR-138, we decided to focus on these molecules in the focal adhesion pathway. An effect on FAK (PTK2) mRNA was observed when the cells were transfected with mimics. At the FAK, protein level seemed to be altered by both mimics and inhibitors of miR-138, in opposite directions (**Figure 8**).

## Discussion

This study shows that expression of miR-138 displays a marked heterogeneity, and it is detectable in a subset of OSCC lesions only. Although performed on a limited number of cases, insufficient for a definitive conclusion, this study points towards a trend for decreased miR-138 expression in tumor tissue when compared to normal oral epithelium. The absence of miR-138 staining in a relatively higher percentage (83%) of tumor samples compared to NHOM (50%) might be an indication for a tumor-suppressive role of miR-138. Inconsistent with this might be the finding that when present, in few cases, the expression of miR138 was increased in both tumor cells and CAFs compared to normal/peritumor regions. Nevertheless, increased expression was associated with lower recurrence and less depth of invasion, indicative again for a tumor-suppressive role. Of note, in our cohort, there was also no specific pattern of association of miR-138 expression with tumor stage, lymph node involvement, or later distant metastasis. This might be due to the relatively low number of cases we have studied, but analysis of the TCGA data set, which comprises many more cases, did not show either any specific association of miR-138 to clinical parameters in OSCC. Taken together, these data do not support a biomarker role for miR-138 in OSCC. However, there are indications for a tumor-suppressive function for miR-138 from both previous and current studies, and thus miR-138 might be of biological importance for a subset of OSCCs.

The heterogeneity of miR-138 expression observed in stroma of OSCC tissues was paralleled by a marked heterogeneity detected in cultured fibroblasts, which might have been even more increased due to selection of different sub-populations of fibroblasts during isolation in culture. We could not identify, however, indications for selective isolation of a certain sub-population over the other. Of importance, despite its heterogenous regulation in fibroblasts, with no clear trend between CAFs and NOFs, increased expression of miR-138 using miR-138 mimics in both CAFs and NOFs had a remarkable effect on their ability to migrate in 3D, to contract collagen gels, and to induce OSCC invasion. Therefore, this study shows that regardless of type of fibroblasts used (CAFs or NOFs), ectopic expression of miR-138 in the fibroblasts results in a consistent inhibition of migration of fibroblasts themselves and of invasion of the adjacent OSCC cells. These findings are in line with the literature suggesting a tumor-suppressive function for miR-138, but while the previous studies addressed the role of miR-138 expressed in tumor cells (26, 27), here we show for the first time a tumor-suppressive effect for miR-138 expression in stromal fibroblasts. In an attempt to understand the mechanism by which alteration in miR-138 expression in the fibroblasts modulates the invasion capabilities by OSCC cells, a couple of functional assays were performed. A crucial effect of increased miR-138 expression in fibroblasts was the morphological transition from spindle-shaped CAFs and NOFs into a stellar morphology, accompanied by a decrease in their motility. The effect on motility might be the underlying mechanism by which decreased miR-138 expression in CAF decreased invasion of adjacent OSCC cells, since CAFs have been shown previously to “lead” the invasion of adjacent OSCC cells (6), and changes in their motility were reflected directly into the invasion ability of adjacent OSCC cells (5).

Similar morphological and motility changes have been previously reported to be associated to loss of FAK function, which regulates focal adhesion assembly and disassembly required for cell motility (33, 34). Our study indicates that FAK was regulated in OSCC-derived CAFs by miR-138, suggesting therefore that the changes in fibroblast morphology and motility observed to occur with increased miR-138 expression might be mediated, among other molecules, *via* FAK. However, these results need to be further confirmed by phenotype rescue experiments to prove that the motility effects we observed after modulation of miR-138 expression are mediated *via* focal adhesion kinase axis. The changes in fibroblasts’ morphology and motility were accompanied by other changes in their molecular profile; ectopic expression of miR-138 significantly decreased several CAF-related markers, particularly those on the TGF-β1 pathway. A link between TGFβ1 pathway and fibroblasts’ motility is well established (35). Moreover, cyclin D1 (CCND1), well known to control proliferation of cells, has also been found to be decreased by ectopic expression of miR-138, and it was linked to cell motility. CCND1-deficient mouse embryonic fibroblasts were previously shown to exhibit increased cellular adhesion and decreased motility compared to the wild type (36). CCND1 deficiency was also associated with reduced migration and increased adhesion or focal adhesion substrate by mice bone marrow-derived macrophages (37). This is in line with the changes in fibroblast morphology and motility we observed in our experiments.

FAK (32, 38, 39), ROCK2 (32, 40), and CCND1 (32, 41) have all been previously shown to be direct targets of miR-138; therefore, it is not surprising that we found their expression changed in the fibroblasts that had an altered miR-138 expression. Taken together, our findings suggest that they act in cohort to control fibroblast migration, and that their expression is regulated by miR-138. ITGA11 has been previously associated with the CAF phenotype (42). Our own qRT-PCR data also showed an inverse correlation between the expression levels of miR-138 and ITGA11, which would indicate that ITGA11 is a direct target of miR-138. However, both the inhibitor/mimic experiments and the gene reporter assay could not confirm this hypothesis.

In conclusion, this study supports a tumor-suppressive role for miR-138 in OSCC while expressed in stromal fibroblasts, despite its heterogeneous expression.

## Data Availability Statement

The raw data supporting the conclusions of this article will be made available by the authors, without undue reservation.

## Ethics Statement

Ethical approval was obtained from the regional ethical committee in Norway (West Norway; REKVest 3.2006.2620, REKVest 3.2006.1341). The patients/participants provided their written informed consent to participate in this study.

## Author Contributions

Conceptualization: SR, AJ, DS, and D-EC. Data curation: SR, HP, and HD. Formal analysis: SR and DS. Funding acquisition: D-EC. Investigation: SR, HP, HD, BL, KH, AK, SL, and EN. Methodology: SR, HD, HP, and DS. Project administration: AJ, DS, and D-EC. Supervision: AJ, DS, and D-EC. Validation: SR. Visualization: SR. Writing—original draft: SR. Writing—review and editing: SR, HP, HD, BL, KH, AK, SL, EN, AJ, DS, and D-EC. All authors contributed to the article and approved the submitted version.

## Funding

This work was supported by the Research Council of Norway through its Centers of Excellence funding scheme (Grant No. 22325), the Western Norway Regional Health Authority (Helse Vest Grant Nos. 911902/2013 and 912260/2019), and the Norwegian Centre for International Cooperation in Education (project number CPEA-LT-2016/10106).

## Conflict of Interest

The authors declare that the research was conducted in the absence of any commercial or financial relationships that could be construed as a potential conflict of interest.

## Publisher’s Note

All claims expressed in this article are solely those of the authors and do not necessarily represent those of their affiliated organizations, or those of the publisher, the editors and the reviewers. Any product that may be evaluated in this article, or claim that may be made by its manufacturer, is not guaranteed or endorsed by the publisher.

## Supplementary Material

Supplementary Figure S1 Diagram showing *in silico*Kaplan-Meier plot for the survival functions of the clinicopathological characteristics using Log-Rank or * Breslow test. Only significant parameters (p<0.05) are shown. Stage I and II: early stage. Stage III and V: late stage.

Supplementary Figure S2 Diagram showing *in silico*miR-138 expression in CAF and NOF 48 hours post transfection of respective mimics and inhibitors of miRNAs, and mimics and inhibitor controls (50nM).

Supplementary Figure S3 Diagram showing *in silico* interrogation of miR-138 targets using miRTarBase database indicating focal adhesion as one of the significantly affected pathways. All 102 miR-138 target genes were fed into DAVID KEGG pathway enrichment analysis (Threshold: Count 2, EASE 0.1, Benjamini test).

Supplementary Table S1 Patient history and tumour characteristics of OSCC cohort.

Supplementary Table S2 List of Taqman Assays (ThermoFisher, USA).

Supplementary Table S3 List of antibodies used in western blot.

Supplementary Table S4 Table showing *in silico* interrogation of miR-138 targets using miRTarBase database indicating focal adhesion as one of the significantly affected pathways. All 102 miR-138 target genes were fed into DAVID KEGG pathway enrichment analysis (Threshold: Count 2, EASE 0.1, Benjamini test).
