# Supplementary material for: MicroRNA-138 Abates Fibroblast Motility With Effect on Invasion of Adjacent Cancer Cells
Source: Front Oncol. 2022 Mar 17;12:833582. doi: 10.3389/fonc.2022.833582 (PMC8968121; doi:10.3389/fonc.2022.833582)
Supplement: Supplementary Figure S1 — Diagram showing in silicoKaplan-Meier plot for the survival functions of the clinicopathological characteristics using Log-Rank or * Breslow test. Only significant parameters (p<0.05) are shown. Stage I and II: early stage. Stage III and V: late stage. [file DataSheet_1.docx]

Supplementary Material

# Supplementary Figures and Tables

##
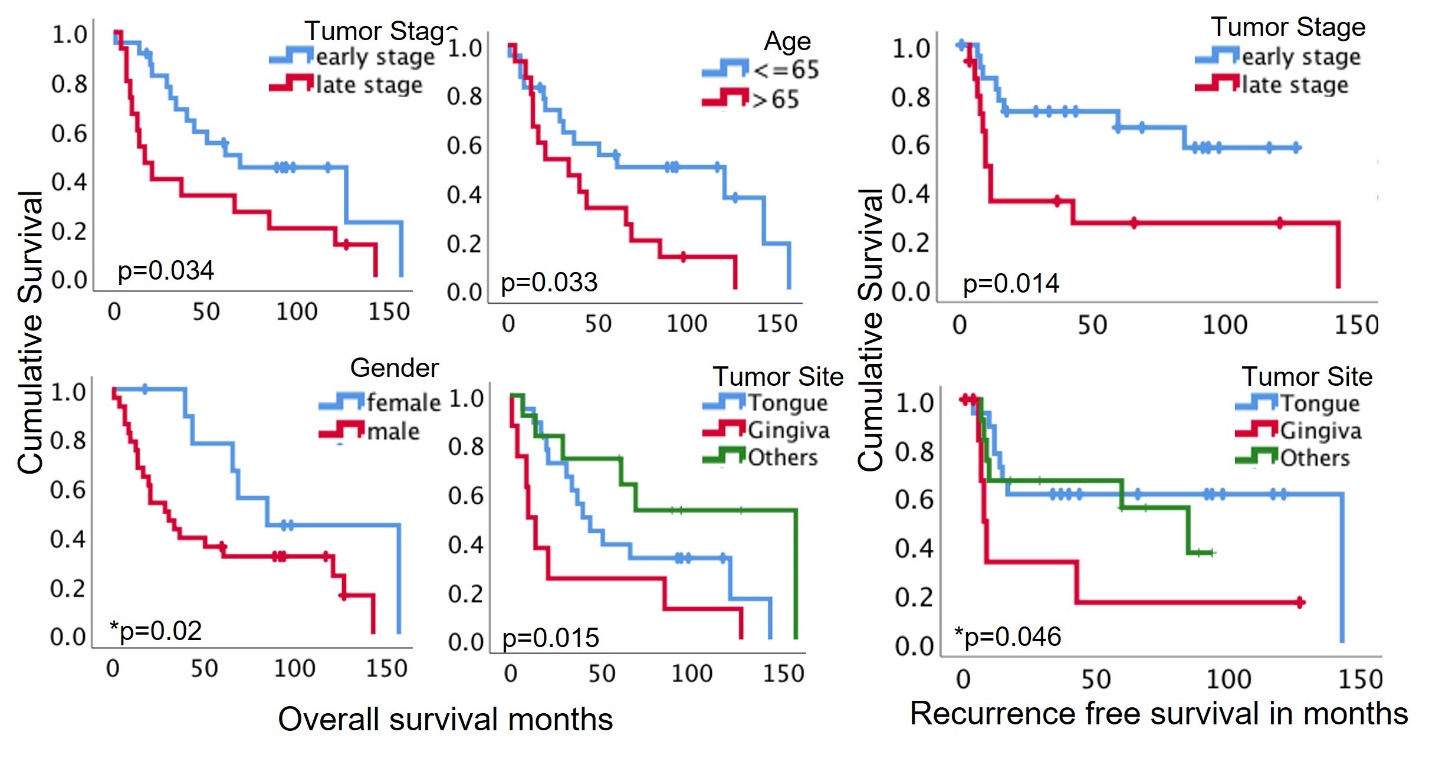
Supplementary Figures


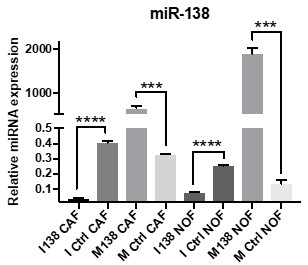
**Fig S1.** Kaplan-Meier plot for the survival functions of the clinicopathological characteristics using Log-Rank or * Breslow test. Only significant parameters (p<0.05) are shown. Stage I&II: early stage. Stage II&IV: late stage.

**Fig S2.** miR-138 expression in CAF and NOF 48 hours post transfection of respective mimics and inhibitors of miRNAs, and mimics and inhibitor controls (50nM).


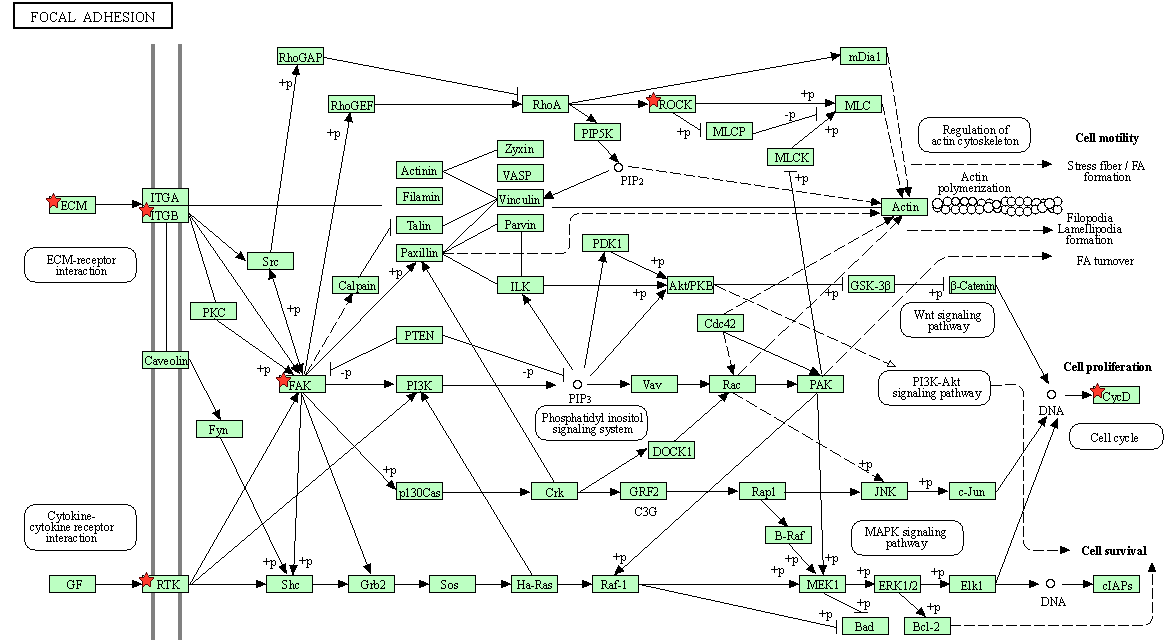


**Fig S3.** In silico interrogation of miR-138 targets using miRTarBase database indicating focal adhesion as a significantly affected pathway. Targeted pathways related to cell motility were selected. Pathways relevant to cancer cells were not selected. All 202 miR-138 – target genes interaction registered in miRTarBase were included in the analysis.

1.2. **Supplementary Tables**

***Table S1:*** *Patient history and tumour characteristics of OSCC cohort*

| Patient population: N=38, Median age: 62, Age range: 34-86 | |
| --- | --- |
| **Parameters N (%)** | **Parameters N (%)** |
| Gender | |
| Female: 10 (26.3)  Median age: 70 | Male: 28 (73.7)  Median age: 62 |
| Smoking | Alcohol |
| No 9 (23.7)  Yes 18 (47.4)  Unknown 11 (28.9) | No- low 10 (26.3)  Moderate- High 12 (31.6)  Unknown 16 (42.1) |
| Tumor site | TNM Stage |
| Tongue 18 (47.4)  Gingiva 8 (21.1)  Buccal 5 (13.2  Floor of mouth 5 (13.2)  Overlapping 2 (5.2) | I 11 (28.9)  II 12 (31.6)  III 5 (13.2)  IV 10 (26.3) |
| Depth of Invasion | *T Stage* |
| Superficial (**<** 4mm) 9 (23.7)  Deep (≥ 4mm) 9 (23.7)  Unquantifiable 20 (52.6) | T1 11 (28.9)  T2 12 (31.6)  T3 6 (15.8)  T4 9 (23.7) |
| Tumor budding score | *N Stage* |
| Low (**<** 5 buds) 18 (47.4)  High (≥ 5 buds) 11 (38.9)  Unquantifiable 9 (23.7) | N0 30 (78.9)  N1 5 (13.2)  N2 3 (7.9)  N3 0 (0) |
| Histological degree of differentiation | *M Stage* |
| Well 32 (84.2)  Poor 5 (13.2) | M0 38 (100)  M1 0 (0) |
| Worst pattern of invasion | Recurrence |
| Type I&II 9 (23.6)  Type III 6 (15.8)  Type IV 23 (60.5) | Yes 19 (50.0)  No 19 (50.0) |
| Distant metastatic progression |  |
| Yes 5 (13.2)  No 32 (84.2) |  |

***Table S2*** *List of Taqman Assays (ThermoFisher, USA)*

| ***Genes*** | ***Taqman Assay ID*** |
| --- | --- |
| hsa-miR-138-5p | 002284 |
| ITGA11 | Hs00201927_m1 |
| PTK2 | Hs01056457_m1 |
| EGFR | Hs01076090_m1 |
| GAPDH | Hs99999905_m1 |
| FAP | Hs00990806_m1 |
| TGFB1 | Hs00998130_m1 |
| AKT1 | Hs00178289_m1 |
| ROCK2 | Hs00178154_m1 |
| 18s | Hs99999901_s1 |
| TGFBR2 | Hs00559660_m1 |
| RPL13A | Hs04194366_g1 |

***Table S3*** *List of antibodies used in western blot*

| ***1^o^ Antibodies*** | ***Cat No*** | ***Dilution*** | ***Protein block*** |
| --- | --- | --- | --- |
| Integrin α11 (1) | - | 1:2000 | 5% Dry milk |
| FAK (D2R2E) | #13009, Cell Signalling | 1:1000 | 3%BSA |
| AKT (pan) (C67E7) | #4691, Cell Signalling | 1:1000 | 3%BSA |
| Beta-Actin | AB8226, Abcam | 1:10000 | 5% Dry milk |

***Table S4*** KEGG Pathways target for miR-138 with miRTarBase

| **S.N** | **KEGG Pathway Term** | **Gene Count** | **P Value** |
| --- | --- | --- | --- |
| 1 | Pathways in cancer | 11 | 2.1E-4 |
| 2 | Transcriptional misregulation in cancer | 7 | 7.8E-4 |
| 3 | p53 signaling pathway | 5 | 9.8E-4 |
| 4 | Proteoglycans in cancer | 7 | 2.0E-3 |
| 5 | MicroRNAs in cancer | 8 | 2.6E-3 |
| 6 | Acute myeloid leukemia | 4 | 6.1E-3 |
| 7 | AMPK signaling pathway | 5 | 8.9E-3 |
| 8 | **Focal adhesion** | 6 | 1.2E-2 |
| 9 | Thyroid cancer | 3 | 1.6E-2 |
| 10 | Hippo signaling pathway | 5 | 1.8E-2 |
| 11 | HIF-1 signaling pathway | 4 | 2.6E-2 |
| 12 | Viral carcinogenesis | 5 | 4.7E-2 |
| 13 | Axon guidance | 4 | 5.3E-2 |
| 14 | Wnt signaling pathway | 4 | 6.4E-2 |
| 15 | PPAR signaling pathway | 3 | 7.4E-2 |
| 16 | PI3K-Akt signaling pathway | 6 | 7.9E-2 |
| 17 | Adherens junction | 3 | 8.1E-2 |
| 18 | Melanoma | 3 | 8.1E-2 |
